# Evaluating the Efficacy of Cervical Tactile Ultrasound Technique as a Predictive Tool for Spontaneous Preterm Birth

**DOI:** 10.4236/ojog.2024.145067

**Published:** 2024-05-24

**Authors:** Vladimir Egorov, Todd Rosen, Jennifer Hill, Meena Khandelwal, Victors Kurtenoks, Brendan Francy, Noune Sarvazyan

**Affiliations:** 1 Advanced Tactile Imaging, Inc., Ewing, New Jersey, USA; 2 Department of Obstetrics, Gynecology and Reproductive Sciences, Rutgers Robert Wood Johnson Medical School, New Brunswick, New Jersey, USA; 3 Department of Maternal-Fetal Medicine, Cooper Medical School of Rowan University, Camden, New Jersey, USA

**Keywords:** Cervical Elasticity, Spontaneous Preterm Birth, Tactile Ultrasound Probe

## Abstract

**Background:**

Premature cervical softening and shortening may be considered an early mechanical failure that predispose to preterm birth.

**Purpose:**

This study aims to explore the applicability of an innovative cervical tactile ultrasound approach for predicting spontaneous preterm birth (sPTB).

**Materials and Methods:**

Eligible participants were women with low-risk singleton pregnancies in their second trimester, enrolled in this prospective observational study. A Cervix Monitor (CM) device was designed with a vaginal probe comprising four tactile sensors and a single ultrasound transducer operating at 5 MHz. The probe enabled the application of controllable pressure to the external cervical surface, facilitating the acquisition of stress-strain data from both anterior and posterior cervical sectors. Gestational age at delivery was recorded and compared against cervical elasticity.

**Results:**

CM examination data were analyzed for 127 women at 24^0/7^ - 28^6/7^ gestational weeks. sPTB was observed in 6.3% of the cases. The preterm group exhibited a lower average cervical stress-to-strain ratio (elasticity) of 0.70 ± 0.26 kPa/mm compared to the term group’s 1.63 ± 0.65 kPa/mm with a p-value of 1.1 × 10^−4^. Diagnostic accuracy for predicting spontaneous preterm birth based solely on cervical elasticity data was found to be 95.0% (95% CI, 88.5 – 100.0).

**Conclusion:**

These findings suggest that measuring cervical elasticity with the designed tactile ultrasound probe has the potential to predict spontaneous preterm birth in a cost-effective manner.

## 1. Introduction

Preterm birth, defined as birth before 37 weeks of gestation, is a significant public health concern. It affects approximately 11% of all live births globally and accounts for more than 1 million infant deaths annually [1] [2]. Spontaneous preterm birth (sPTB) not only results in immediate consequences such as neonatal mortality and morbidity but also has long-term implications including cognitive, behavioral, and neurological deficits in the surviving infants [3] [4]. Consequently, early and accurate prediction of sPTB is of paramount importance to improve neonatal outcomes and reduce healthcare costs [5].

Despite the substantial progress in understanding the pathophysiology of preterm birth, predicting and preventing it remains a significant challenge [6] [7] [8]. Several conventional methods have been employed to identify women at risk of sPTB, including maternal history, clinical examination, biochemical markers, and ultrasound-based cervical length measurement [9] [10]. However, these methods have their limitations, such as low sensitivity and specificity, invasive nature, or the need for skilled personnel [10] [11]. Thus, there is an increasing demand for an accurate, inexpensive, and easy-to-use technique for detection conditions leading to sPTB.

Cervical elastography has emerged as a diagnostic tool for assessing the biomechanical properties of the cervix during pregnancy [12] [13]. The rationale for using cervical elastography to predict preterm birth lies in the significant structural and functional changes that the cervix undergoes throughout pregnancy, including softening, shortening, and dilation, all detectable by elastographic techniques [14]. Several studies have reported an association between cervical stiffness and the risk of preterm birth [15] [16]. Reduced cervical stiffness, as assessed by elastography, has been found to correlate with a higher likelihood of preterm birth [17] [18]. Furthermore, some researchers have demonstrated that cervical elastography may outperform conventional cervical length measurement in predicting preterm birth [19] [20]. However, the optimal elastographic parameters for predicting preterm birth remain uncertain, and the results are often inconsistent and inconclusive [21] [22] [23]. Additionally, this method requires high-end ultrasound imaging equipment, which can be expensive.

To investigate a novel elastographic approach for cervical measurements, a cost-effective Cervix Monitor (CM) device was designed, featuring a tactile ultrasound probe with four tactile sensors and a single ultrasound transducer. A pilot clinical study involving 10 non-pregnant and 10 pregnant women with the CM probe demonstrated clinically acceptable measurement performance and reproducibility. The availability of stress-strain data enabled assessment of cervical elasticity and length [24].

The objective of this study is to evaluate the efficacy of the tactile ultrasound approach in predicting sPTB at 24 – 28 weeks of gestation in women with singleton pregnancies.

## 2. Materials and Methods

### 2.1. Study Design

A prospective observational study was conducted at Rutgers Robert Wood Johnson Medical School (New Brunswick, NJ) and The Cooper Health System (Camden, NJ) between January 2020 and April 2022. The inclusion criteria for the study were: 1) singleton in the current pregnancy, and 2) gestational age from 24 to 28 weeks at the time of Cervix Monitor measurement. The exclusion criteria were: 1) fetal anomaly, 2) history of fetal reduction in the current pregnancy to a singleton gestation, 3) preterm rupture of membranes, 4) current or planned cervical cerclage, 5) planned indicated preterm delivery, 6) active known cancer of the colon, rectum wall, cervix, vaginal, uterus or bladder, 7) ischemic heart disease and/or arrhythmia, and 8) active skin infection or ulceration within the vagina/vulva (herpes infection). The Informed Consent Form and Clinical Protocol were reviewed and approved centrally by the Western Institutional Review Board before the study’s initiation. Demographic data including maternal age, race/ethnicity (NIH requirement), height, recorded weight at time of examination, parity, and prior obstetrical history were recorded at time of informed consent. Maternal comfort level during the CM examination was recorded on a 3-point scale (1 - more comfortable than bimanual examination, 2 - the same, 3 - less comfortable). Mode of delivery and gestational age at birth were extracted from the electronic database at a later date.

**Figure 1.**
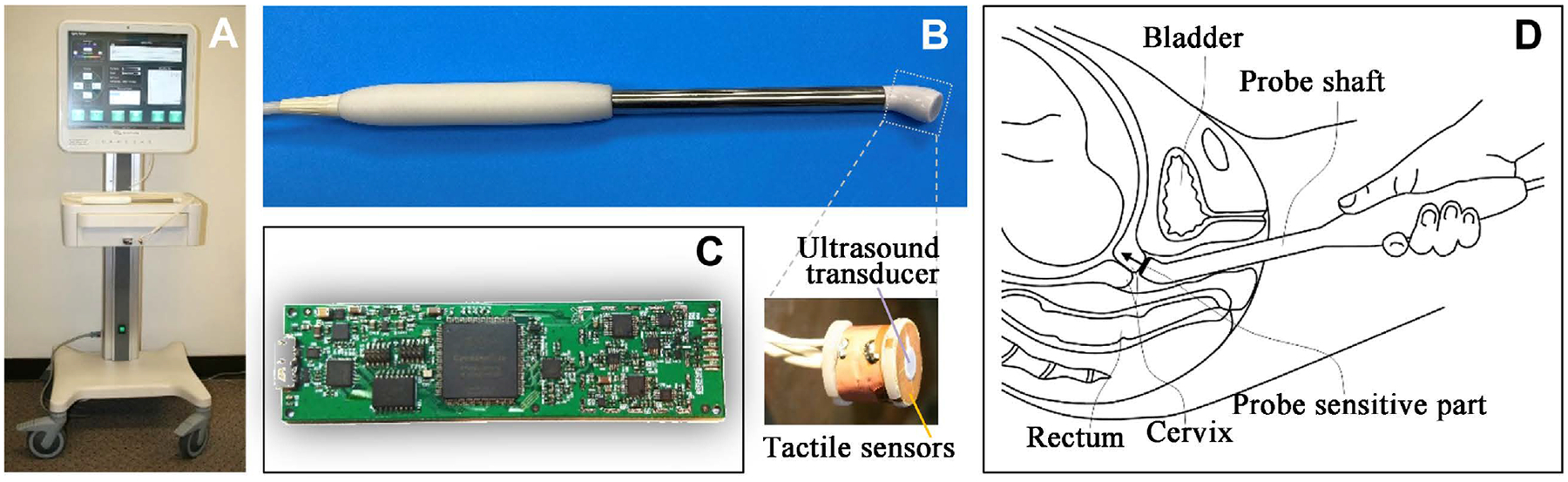
Cervix Monitor design and application. (A) perspective view of the device, (B) cervical probe featuring a single ultrasound transducer and four tactile sensors, (C) printed circuit board designed for data acquisition from both ultrasound and tactile sensors, and (D) cervical probe positioning during data acquisition; the arrow indicates the direction of cervical deformation during measurement.

This study was conducted in two phases: development and validation. In the development phase, the CM examination procedure was optimized and ultrasound B-mode images with the CM probe were recorded for the cervix during the CM probe application to verify anatomical locations of the probe during measurements. The CM probe, shown in Figure 1B, was used in the validation study.

### 2.2. Cervix Monitor

The CM was designed as a cart-based device (Figure 1A) featuring a medical-grade touchscreen computer and a detachable cervical probe (Figure 1B). The CM probe comprises a tactile array with four capacitive pressure sensors surrounding an ultrasound transducer, as illustrated in Figure 1B. The probe’s sensitive plane was angled by 40 degrees from the basic probe axis (see Figure 1D).

The ultrasound 5.0 MHz transducer operates in pulse-echo mode with a data acquisition resolution of 20 ns (50 MHz sample rate) and has a diameter of 3.0 mm. Biocompatible, two-component silicones were used to ensure sensor assembly functionality, durability, stability, and mechanical protection. The pressure measurement noise level was below 30 Pa within the operational range of 30 kPa. The ultrasound transmitting pulses had a peak amplitude below 50 V and a length less than 1 μs, providing acoustic power significantly below the FDA-established limits for ultrasound emission in obstetrics: spatial-peak temporal-average (I_spta_) of 13 mW/cm^2^, spatial-peak pulse-average intensity (I_sppa_) of 86 W/cm^2^, and mechanical index (MI) of 1.0. Medical-grade 316 stainless steel, commonly used in the production of surgical instruments, was employed to fabricate the probe body (see Figure 1B). A proprietary printed circuit board (see Figure 1C) was designed to perform the dual functions of tactile signal acquisition and generation/acquisition of synchronized ultrasound signals. Its key features include serving four tactile/pressure sensors and one ultrasound transducer at 100 data frames per second. Important to note that no expensive components were used in the CM probe and electronics shown in Figure 1.

A custom-designed linear array of 32 ultrasound transducers (Vermon, France) was used in the same cervical probe to image the cervical tissue around the probe in B-mode with ArtUs electronics (Telemed, Vilnius, Lithuania) during the development study.

The CM software interface enabled real-time observation of the cervical ultrasound signal and the applied pressure. The cervical length was calculated from the time-of-flight of an ultrasound pulse reflected from the internal cervical surface; similar to used technique in the feasibility study [24] [25] [26]. The ultrasound peak position for the cervical internal surface was detected using a signal envelope after Gaussian complex wavelet filtering [27] in C++. Cervical elasticity was calculated as the ratio of the applied load (stress) on the cervical surface from the CM probe to the resultant changes in cervical length (strain). This approach was optimized with soft silicone tissue models with known mechanical parameters in bench testing and verification.

### 2.3. Examination Procedure

The CM examination procedure included: 1) placing the patient in the lithotomy position; 2) inserting the speculum into the vagina to ensure proper visualization and access to the cervix; and 3) performing CM measurements at 12 and 6 o’clock positions with real-time observation of the applied pressure level and capturing the reflected ultrasound signal. The applied cervix pressure was limited to 30 kPa (<2.5 N to the probe tip) during CM measurements.

### 2.4. Statistical Analysis

General descriptive statistics (mean values, standard deviations, boxplots) and *p*-values for two-sample *t*-tests were calculated using MATLAB version R2022b (MathWorks, MA). Receiver operating characteristic (ROC) analysis was performed using MedCalc 2023 software (MedCalc Software Ltd, Belgium). The method of DeLong *et al*. [28] was employed for the calculation of the 95% confidence intervals for ROC analysis.

## 3. Results

Overall, this study enrolled 166 women. Five subjects did not attend the CM examination, seven were lost to follow-up, and two cases were excluded due to an operator error in CM data recording. The development study included 34 patients, while the validation study involved 132 patients. Among these 132 cases, spontaneous preterm birth occurred in eight cases and five preterm births in five women were due to clinically indicated maternal or fetal conditions.

In the subsequent data analysis, the CM examination data for 127 women at 24^0/7^ - 28^6/7^ gestational weeks were utilized. Table 1 presents basic characteristics for the spontaneous preterm group of eight subjects and the term group of 119 subjects. The mean maternal age, subject’s height, weight, and gestational age at CM examination showed no statistically significant differences between both groups (Table 1).

The mean gestational age for the preterm group was 34 weeks and five days, whereas for the term group, it was 39 weeks and one day. There was no statistically significant difference between these groups in terms of cervical length (p-value = 0.11). The preterm group demonstrated lower cervical stress-to-strain ratio (elasticity) values in both the anterior and posterior cervical compartments, with average values across these compartments being 0.70 ± 0.26 kPa/mm, compared to 1.63 ± 0.65 kPa/mm for the term group; this difference was statistically significant with a p-value of 1.1 × 10^−4^.

**Figure 2.**
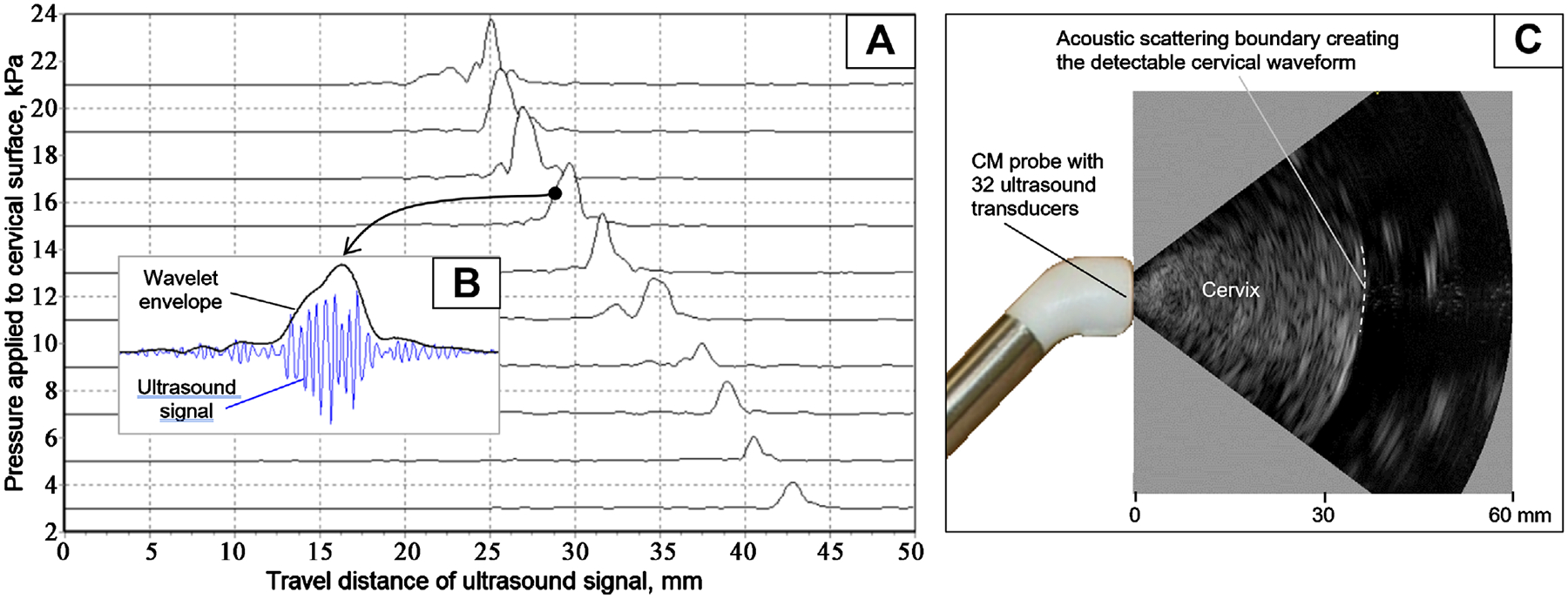
Ultrasound cervical waveforms recorded from a woman at 25 gestational weeks. (A) wavelet envelopes of ultrasound signals reflected from the internal cervical surface during cervical compression by the CM probe, (B) zoomed view of the wavelet envelope showing the detectable ultrasound signal, and (C) sagittal cervical sector image acquired with a probe similar to the used in CM, but having a phased ultrasound array.

Figure 2 illustrates the acquisition of return acoustic signals from the internal cervical surface during the application of measured pressure (see vertical axis in Figure 2A) from the probe to the cervical external surface. As shown, the cervix has been compressed from 43 mm to 25 mm at an applied pressure of 21 kPa. Figure 2B presents the original ultrasound waveform with an envelope calculated using a complex Gauss wavelet at 5.0 MHz. To ensure that we capture the signal from the internal cervical surface, we incorporated an ultrasound array in the same probe, which provided B-mode imaging (see Figure 2C).

**Figure 3.**
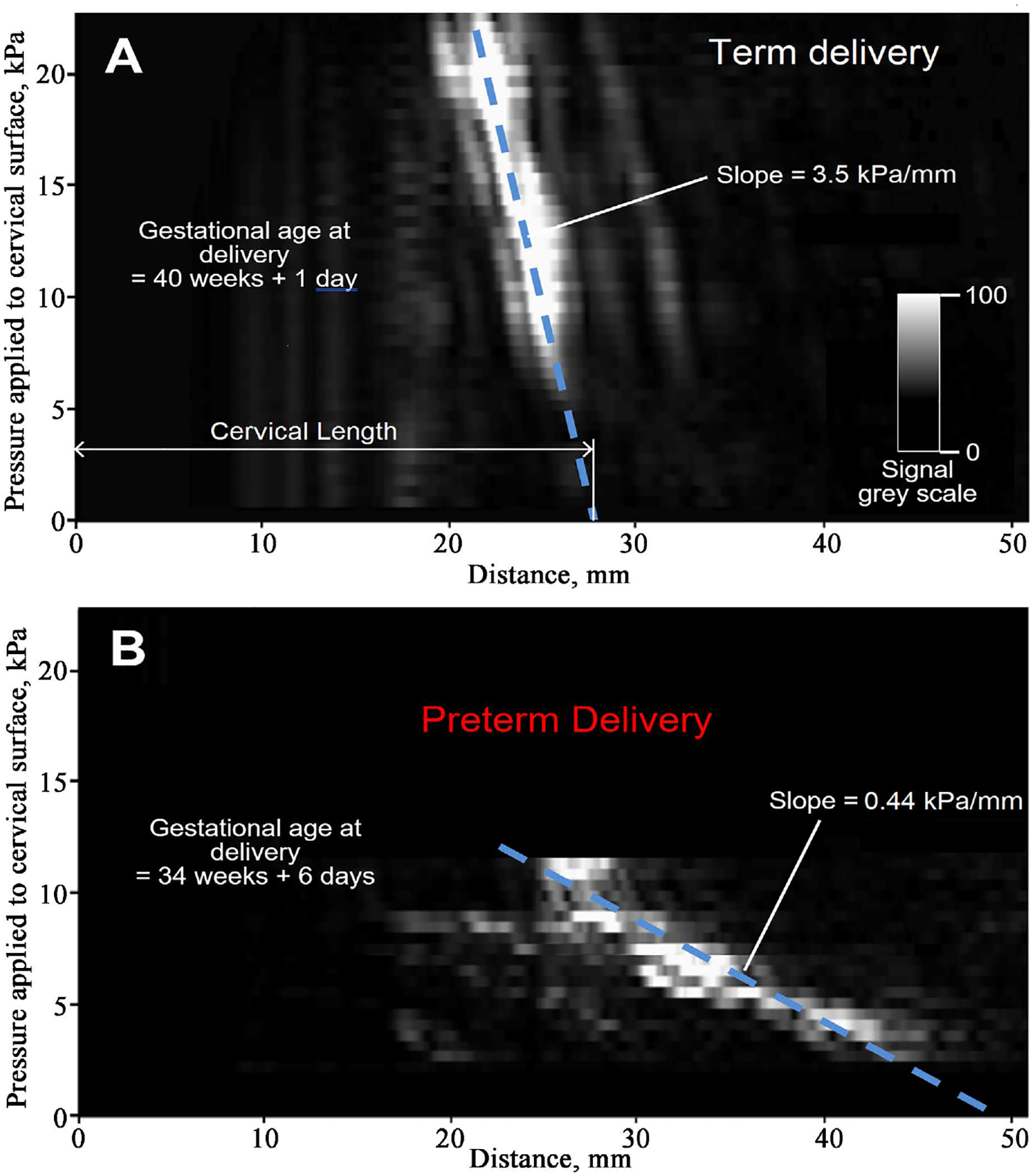
Stress-strain cervical mapping using ultrasound signals reflected from the internal cervical surface and applied pressure at cervix by the CM probe, acquired at 24 – 28 gestational weeks. (A) term birth at 40 and 1 day, (B) preterm birth at 34 weeks and 6 days.

Figure 3 presents two stress-strain cervical maps for two subjects to illustrate the method used to calculate the stress-to-strain ratio as a slope. The horizontal axis represents the distance traveled by ultrasound signals reflected from cervical tissue. The vertical axis displays the pressure value applied to the cervical surface by the CM probe. The amplitude of the reflected ultrasound signals in these maps is presented by a linear grayscale, as shown in Figure 3A. In these maps, the white color corresponds to a higher amplitude of the reflected ultrasound signal from the internal cervical surface. The slope, marked by the dashed blue line in these examples, changes from 3.5 kPa/mm in Figure 3A (term birth) to 0.44 kPa/mm in Figure 3B (preterm birth). The prolongation of the slope line to its intersection with the horizontal axis (see Figure 3A) was used for calculating cervical length in this study.

**Figure 4.**
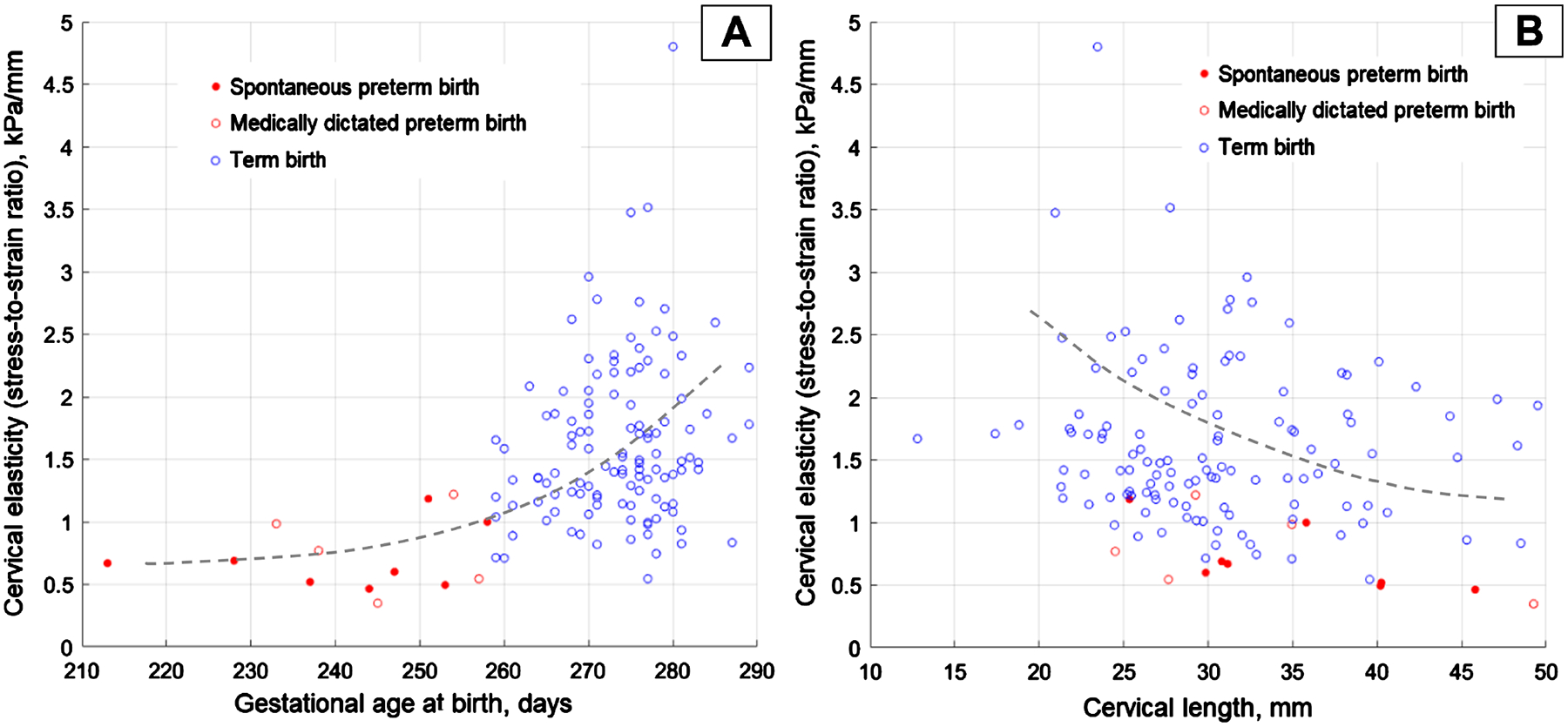
CM examination data acquired at 24 – 28 gestational weeks. (A) cervical elasticity versus gestational age at birth and (B) cervical elasticity versus cervical length.

Figure 4A displays the CM-measured cervical elasticity at 24 – 28 gestational weeks versus gestational age at birth. Figure 4B shows the CM-measured cervical elasticity versus CM-measured cervical length. The data presentation in Figure 4B is provided because a tendency for decreased cervical elasticity is observed with an increase in cervical length (see dashed line).

**Figure 5.**
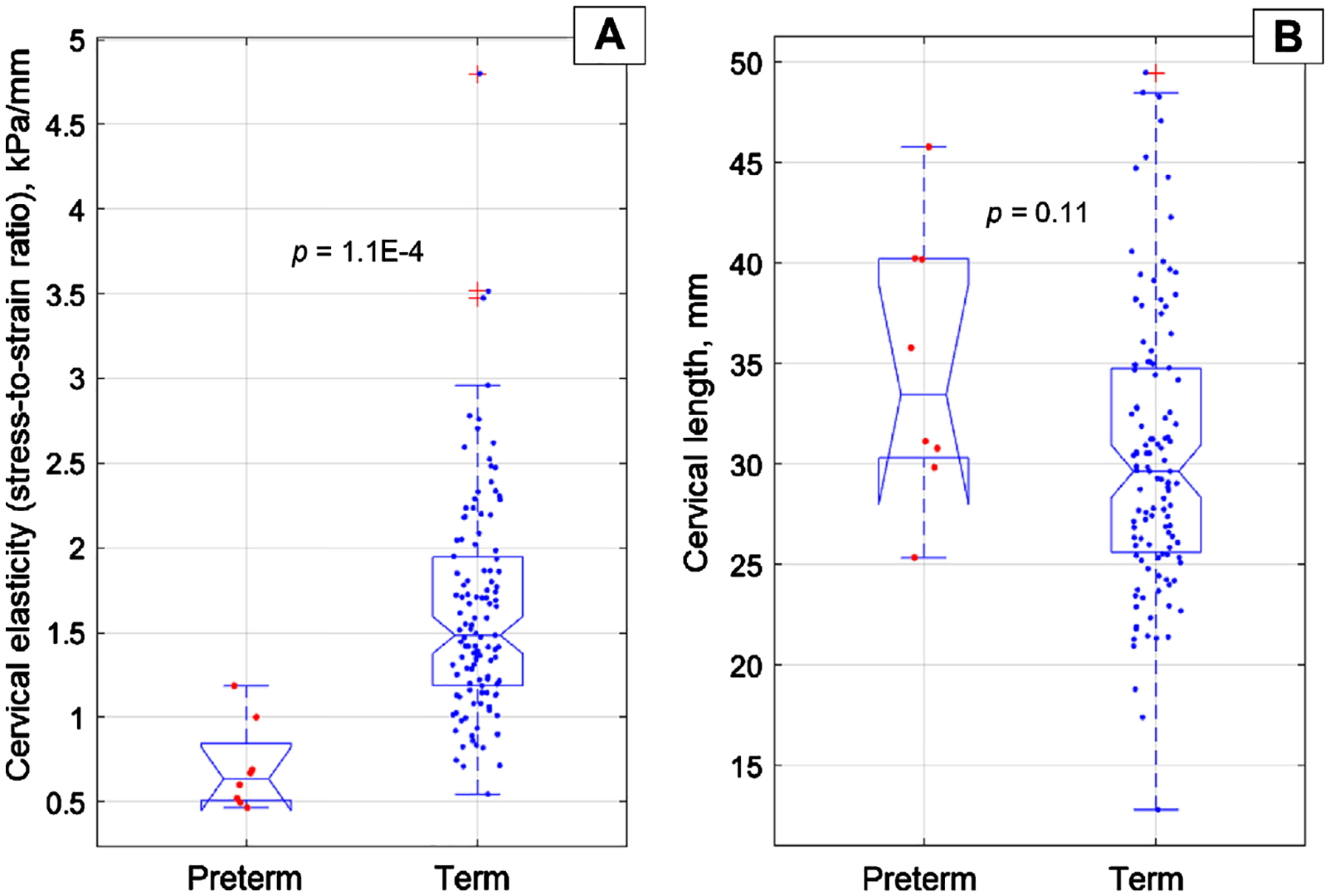
Boxplots for CM examination data acquired at 24 – 28 gestational weeks.(A) cervical elasticity, and (B) cervical length for preterm and term birth groups.

Figure 5 presents boxplots to compare the average cervical elasticity and length for the preterm and term groups. The boxplot in Figure 5A demonstrates a significant decrease in cervical elasticity in the preterm group with a *p* = 1.1 × 10^−4^. However, for cervical length, no statistically significant difference was found (see Figure 5B) with a *p*-value of 0.11.

**Figure 6.**
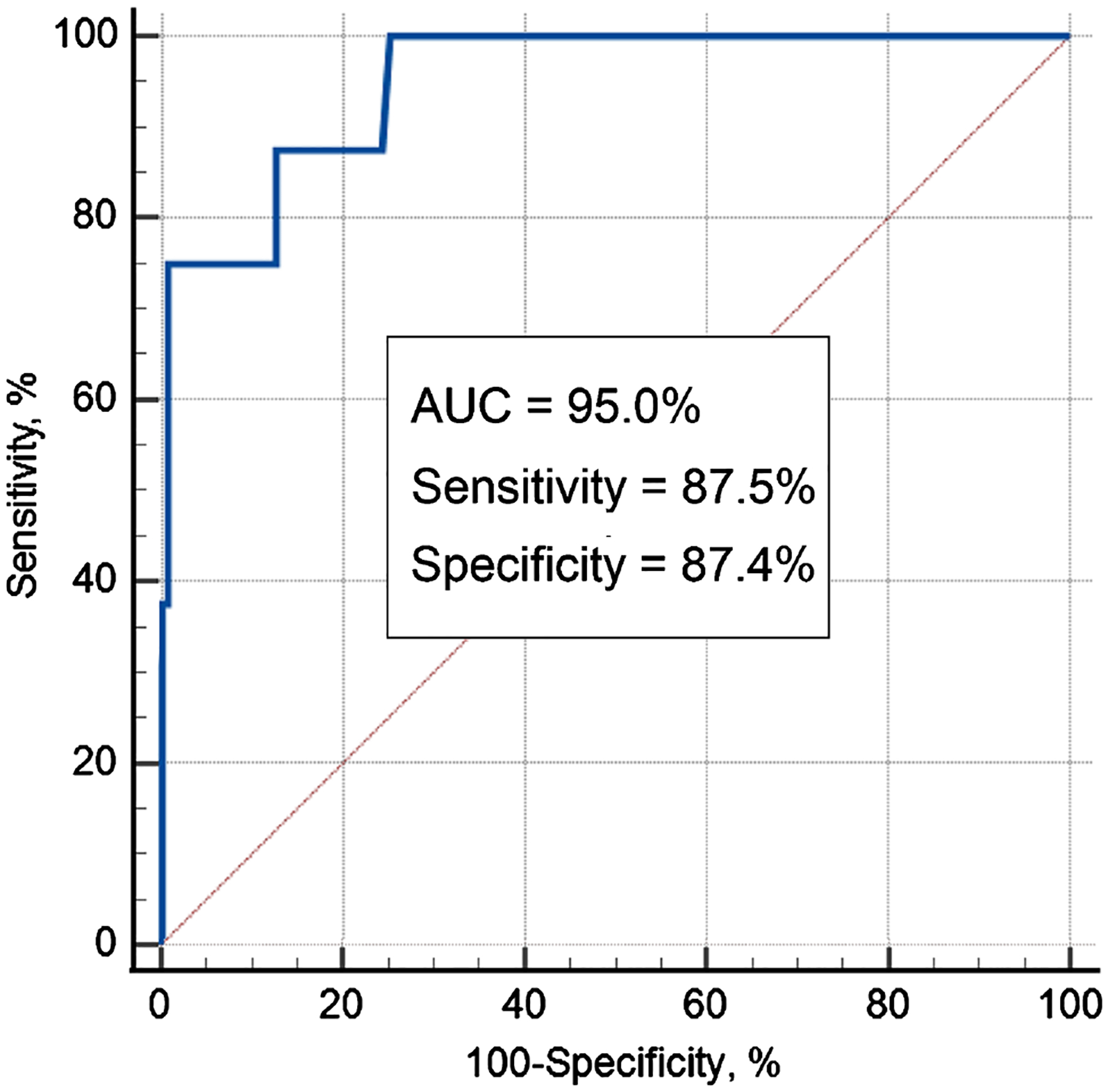
ROC curve for prediction of spontaneous preterm birth (<37 weeks) resulting from cervical elasticity data acquired at 24 – 28 gestational weeks with the CM.

Receiver operating characteristic (ROC) analysis revealed that average cervical elasticity had a sensitivity of 87.5% (95% CI, 47.3 – 97.7) and a specificity of 87.4% (95% CI, 80.1 – 92.8) for predicting sPTB at a cutoff value of 1.0 kPa/mm for the cervical stress-to-strain ratio (average for anterior and posterior compartments). The area under the ROC curve (AUC) was 95.0% (95% CI, 88.5 – 100.0; *p* = 1.1 × 10^−4^), indicating a high predicting capability of cervical elasticity data (stress-to-strain ratio) for sPTB (see Figure 6).

The maternal comfort level during the CM examination was reported by 54 women as more comfortable than the bimanual examination, by 65 women as the same, and by 8 women as less comfortable.

## 4. Discussion

This study involving 127 women, including 8 spontaneous preterm and 119 term births, demonstrated that the tactile ultrasound probe may predict sPTB at 24 – 28 gestational weeks with an accuracy of 95.0% (95% CI, 88.5 – 100.0). A key outcome of this research hinges on measuring cervical elasticity, expressed as a stress-to-strain ratio.

No statistically significant difference was found between the preterm and term groups regarding cervical length (*p*-value = 0.11). Moreover, a reverse tendency compared to that reported in the literature [7] was observed. We noted an increase in cervical length in the sPTB group as measured with the CM probe (see Figure 5B). One possible explanation for this increase is that the CM probe’s pressure measurement is overly sensitive to initial contact with the cervical surface, whereas the conventional ultrasound probe may easily deform the soft cervix, leading to errors in length measurement. Anyway, the cervical length measurement with this CM probe requires more exploration.

Another finding in this study is that cases of medically dictated preterm births demonstrated decreased cervical elasticity similar to spontaneous preterm birth cases (see five red hollow circles in Figure 4A). This may be explained by the reproductive system of women adjusting to unfavorable maternal and/or fetus conditions, preparing for possible preterm birth, which includes cervical softening.

We identified 17 published studies focusing on predictive capabilities of cervical ultrasound for sPTB. Among them, nine studies utilized strain elastography (SE) [15] [17] [18] [19] [27] [28]–[37] and eight employed shear wave elastography (SWE) [22] [23] [29] [30] [33] [34] [35] [36]. Sixteen of 17 studies concluded that cervical elastography could predict preterm birth. However, one study, involving 10 preterm cases, found no statistically significant differences in cervical elasticity between preterm and term births [22]. Four studies reported results in terms of Odds Ratio (OR), ranging from 1.15 to 6.5 [15] [23] [28] [30]. In the studies that characterized sensitivity, specificity, and accuracy, these values ranged from 59% to 96.7%, 57.9% to 96.3%, and 63.4% to 98%, respectively. Our current study contributes to the growing body of evidence that cervical elasticity is a meaningful predictor of preterm birth, potentially more effective than measurements of cervical length by transvaginal ultrasound, which is currently in widespread clinical use. Additionally, it demonstrates the applicability of directly measured cervical stress-to-strain ratios using a simplified tactile ultrasound probe in predicting sPTB.

Assessments of the maternal serum proteome and transcriptome early in pregnancy have demonstrated that some cases of sPTB result from events that occur well before a woman manifests symptoms of preterm labor [38] [39]. In the current study, we have found that changes in cervical elasticity represent another detectable change occurring before the clinical syndrome of preterm labor becomes apparent. This discovery provides an additional opportunity for early intervention to prevent preterm birth.

Combining cervical elasticity, which has now been demonstrated to predict preterm birth in multiple prospective studies, with other indicators, may enhance the assessment of sPTB risk. Preterm labor can result from multiple pathological processes [40], yet most high-risk sPTB women are treated similarly. By integrating assessment of a patient’s history, serum protein or RNA markers, and cervical secretions through tests such as fetal fibronectin [41], and considering cervical elasticity, a more personalized treatment approach to prevent sPTB could be developed. This approach may lead to improved outcomes in addressing this significant clinical challenge.

It is evident that larger, multi-center studies are required to validate the CM examination procedures and establish cutoff values for cervical elasticity, preferably using absolute units. An unanswered question remains regarding the distribution of cervical elasticity along the cervical canal, which warrants further investigation. Additionally, translating the stress-to-strain ratio into the Young’s modulus scale is another area of inquiry. This aspect is currently being explored through finite element modeling and validation with cervical tissue models; however, it is beyond the scope of this article.

### Study strength:

Despite its relatively small sample size, the strength of this study is evident in the CM technique’s ability to yield a 95% confidence interval of 88.5% – 100.0% for detecting cervical conditions leading to sPTB. A major advantage of the CM device is its potential as a cost-effective alternative to SE and SWE ultrasound devices, which require high-quality imaging capabilities. The probe could potentially have a disposable intravaginal measuring part, which costs below $100.

### Study weakness:

The cervical length measurements with the CM probe may provide overestimated values due to initial cervical deformation and inconsistently low-amplitude ultrasound signals, especially in softer cervixes. Secondly, the study’s sample size did not allow the exploration of potential racial and ethnic differences in cervical elasticity cutoff for sPTB. Thirdly, this study does not assess causes of preterm birth through clinical suspicion or placental pathology, which represent another limitation. Future studies involving larger and diverse populations are needed to address these limitations.

## 5. Conclusion

The designed tactile ultrasound probe offers a novel and cost-effective approach for characterizing cervical elasticity and demonstrates promising potential for predicting spontaneous preterm birth. This innovation not only enhances diagnostic capabilities but also provides a more accessible option for healthcare providers. By potentially improving management and prevention strategies, it could contribute significantly to saving children’s lives.

## Figures and Tables

**Table 1.** Subject’s demography, mean and *p*-values (*t*-test) for the preterm versus term groups.

Characteristics	Spontaneous preterm delivery < 37 weeks (n = 8)	Term delivery ≥ 37 weeks(n = 119)	*p*-value
Maternal age, years	32.0 ± 5.3	30.8 ± 6.2	0.58
Height, cm	161.5 ± 8.4	162.7 ± 6.7	0.96
Weight, kg	82.7 ± 13.8	81.1 ± 17.9	0.80
Parous women	4 (50%)	45 (38%)	-
Prior history of PTB	2 (25%)	11 (9%)	-
Gestational age at CM examination, weeks + days	27 + 1	26 + 4	0.32
Gestational age at birth, weeks + days	34 + 5	39 + 1	4.7 × 10^−22^
Anterior cervical length, mm	34.9 ± 9.8	30.8 ± 8.5	0.19
Posterior cervical length, mm	34.8 ± 8.8	30.1 ± 7.2	0.08
Average anterior and posterior cervical length, mm	34.9 ± 7.7	30.4 ± 6.8	0.11
Anterior cervical elasticity (stress-to-strain ration), kPa/mm	0.73 ± 0.44	1.67 ± 0.89	3.6 × 10^−3^
Posterior cervical elasticity (stress-to-strain ration), kPa/mm	0.68 ± 0.21	1.58 ± 0.59	3.0 × 10^−5^
Average anterior and posterior cervical elasticity (stress-to-strain ration), kPa/mm	0.70 ± 0.26	1.63 ± 0.65	1.1 × 10^−4^
